# Supplementary material for: Synchronized seasonal excretion of multiple coronaviruses coincides with high rates of coinfection in immature bats
Source: Nat Commun. 2025 Jul 17;16:6579. doi: 10.1038/s41467-025-61521-7 (PMC12271399; doi:10.1038/s41467-025-61521-7)
Supplement: Supplementary file 1 — Supplementary Information [file 41467_2025_61521_MOESM1_ESM.pdf]

Supplementary information

for

Synchronized seasonal excretion of multiple coronaviruses coincides with high rates of coinfection in immature bats

Alison J. Peel, Manuel Ruiz-Aravena, Karan Kim, Braden Scherting, Caylee A. Falvo, Daniel E. Crowley, Vincent J. Munster, Edward J. Annand, Karren Plain, Devin N. Jones, Tamika J. Lunn, Adrienne S. Dale, Andrew Hoegh, John-Sebastian Eden, Raina K. Plowright

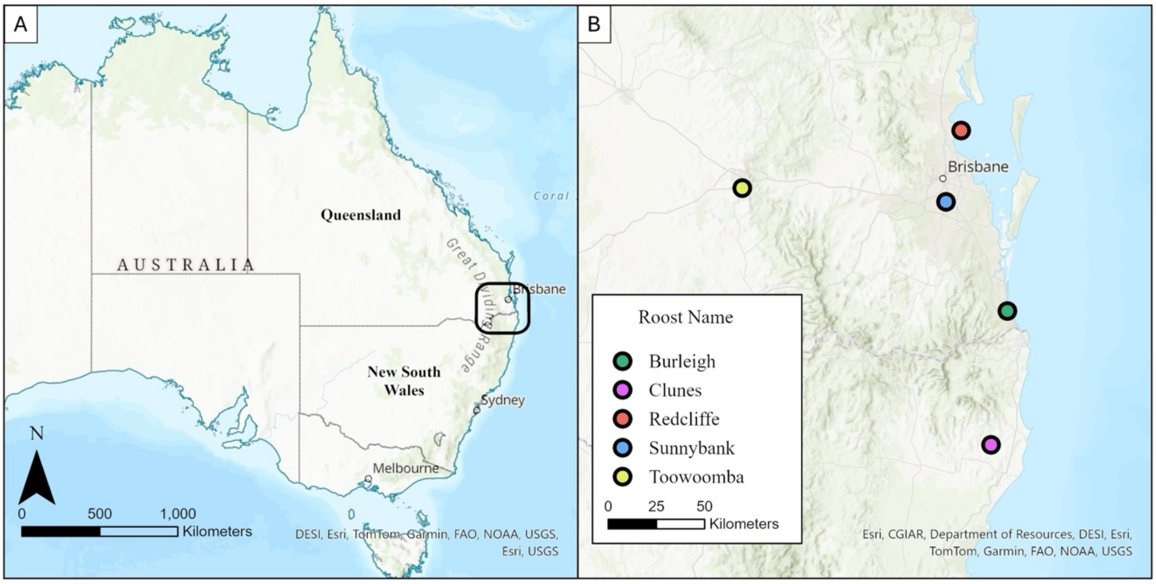

SI Figure 1. The Study Area. A: Map of Australia, with the study area highlighted by the black box. B: Study area, showing five study sites (Four in the state of Queensland, one in the state of New South Wales). Maps were created using ArcGIS Pro software by Esri. The basemap attribution is included within each figure panel.

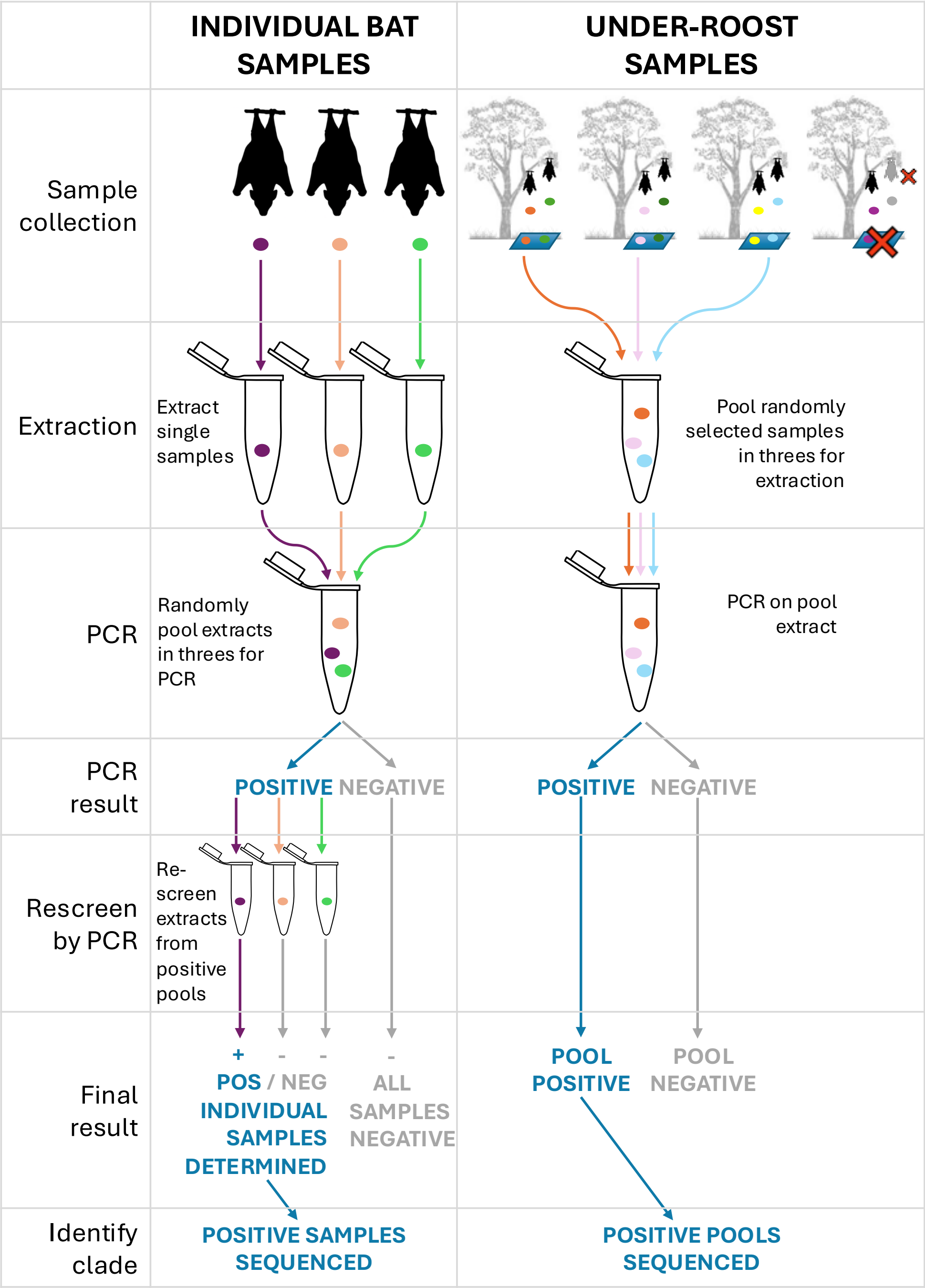

SI Figure 2. Model-guided screening framework. Individual bat samples: RNA was extracted from each fecal sample separately, then extracts pooled in groups of three within the same sampling session for coronavirus PCR. For individual bat pools testing PCR-positive, component samples were rescreened individually to identify specific positive individuals, and subsequently sequenced to identify clades. B) Under-roost samples: From sheets where only black flying foxes were recorded as roosting overhead, 30 samples were randomly selected and pooled to optimize cost efficiency for extraction (three samples per pool). Samples within pools were shuffled to prevent pools containing multiple samples from identical sheets. Under-roost pool extracts were screened using the coronavirus PCR and, following our model-guided approach to maximize information gain relative to cost, classified as positive or negative without component rescreening. All positive pools were sequenced to determine clades.

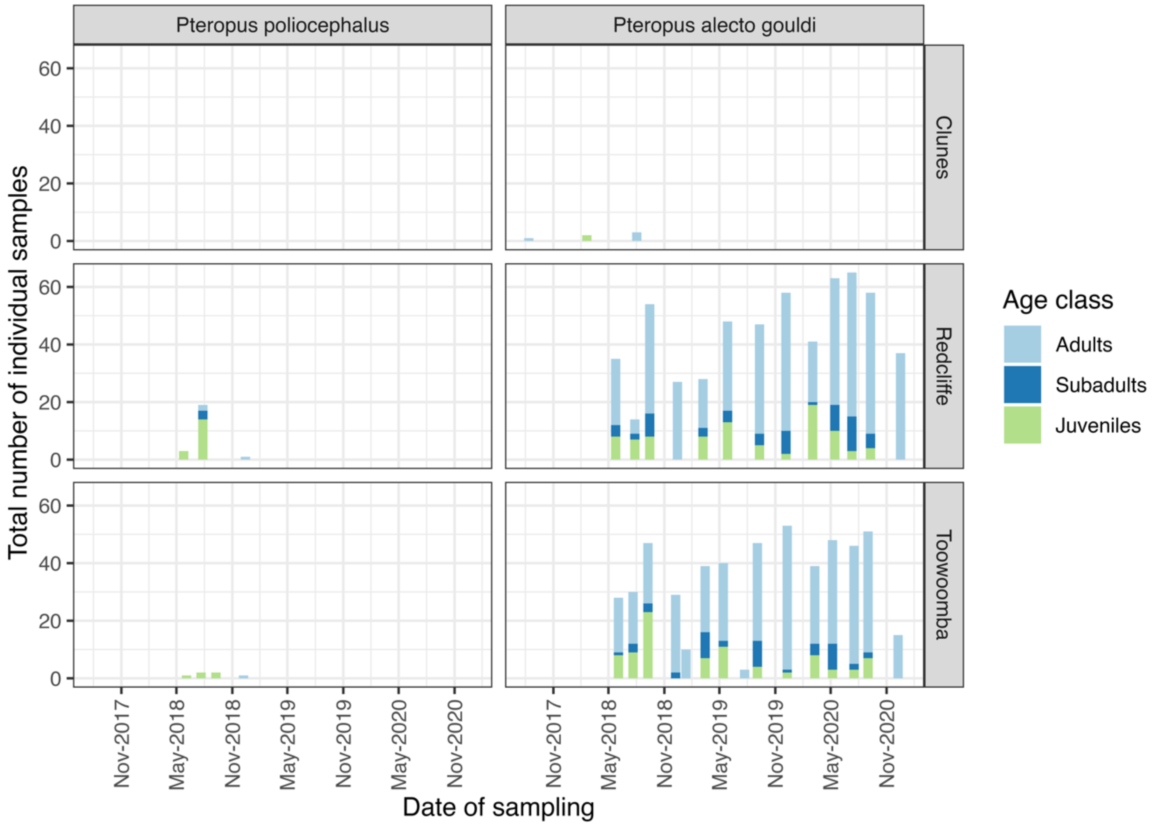

SI Figure 3. Catching sampling effort over time by site bat species (right column: black flying fox, BFF, *Pteropus alecto gouldi*; left column: grey-headed flying fox, GHFF, *Pteropus poliocephalus*), and age categories (Juvenile (estimated <12 months), subadult (estimated 1 – 2 years), adult (estimated > 2 years)). Sample sizes are provided in SI Table 1.

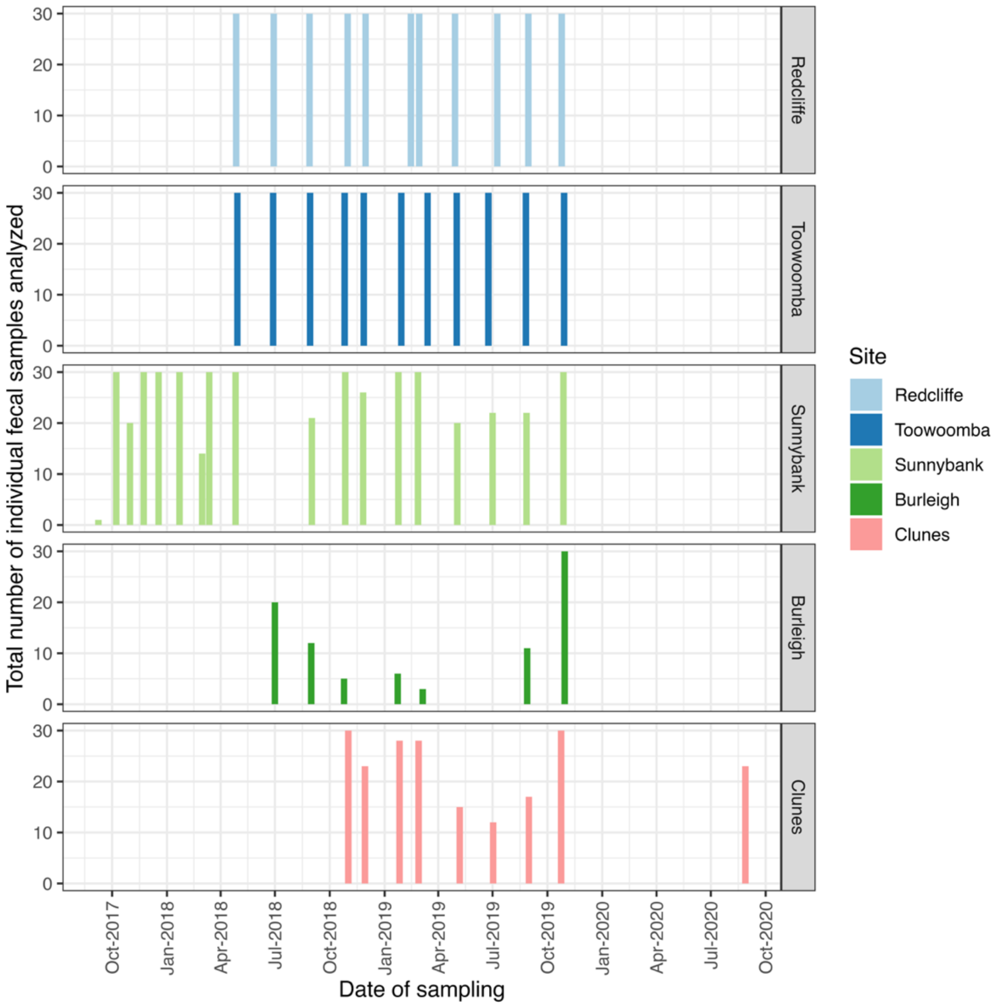

SI Figure 4. Effort of population-level (underroost) sampling across all sites, showing the total number of samples collected, and subsequently pooled for testing. Sample sizes are provided in SI Table 2.

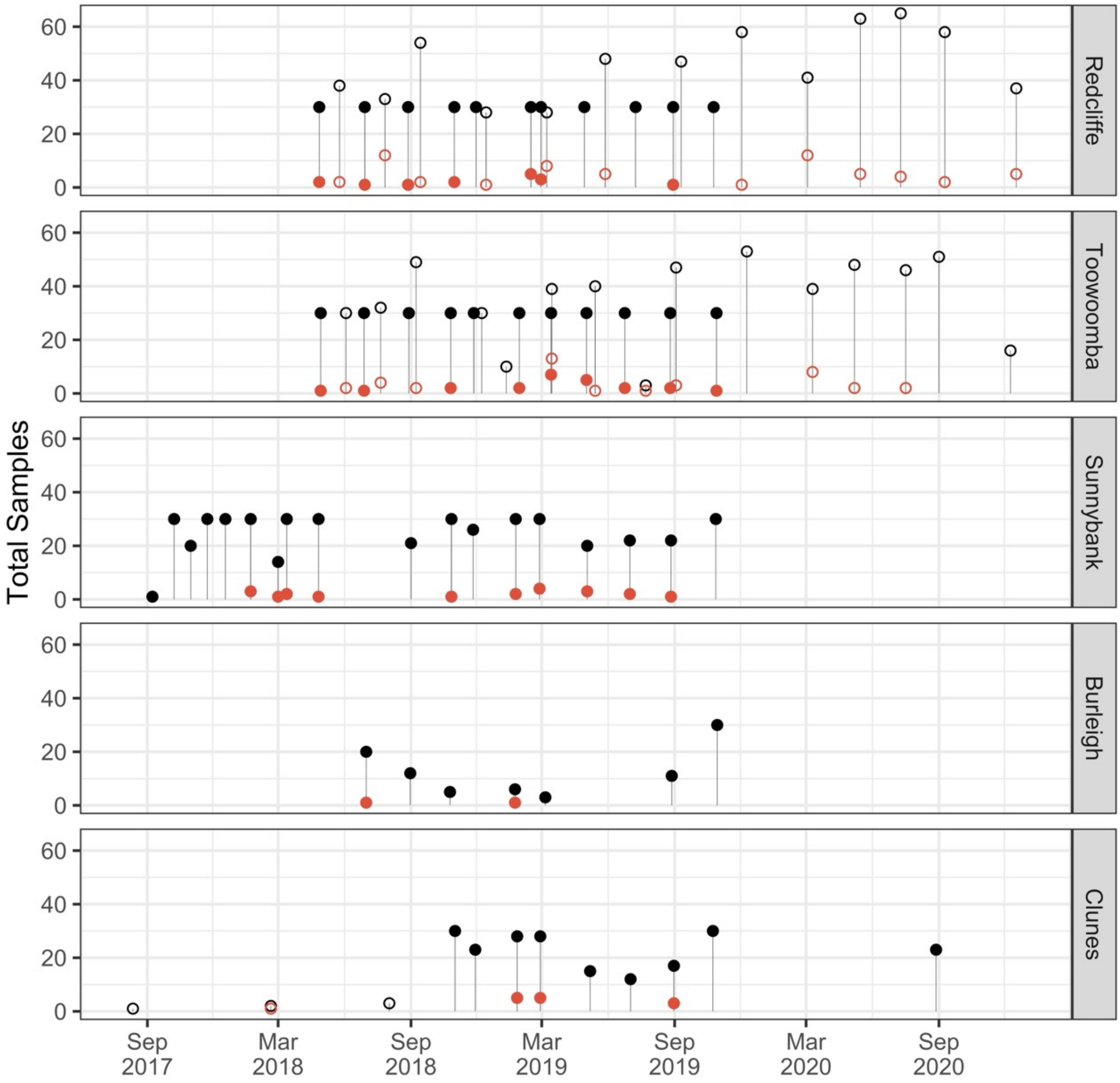

SI Figure 5. Number of samples and pools tested and positive for coronavirus RNA over time, across 5 sites. Open circles represent samples from individuals and closed circles represent under-roost samples that were combined into pools, generally of size 3. Black coloring represents the total number of samples tested for a particular date and red represents the number of positive coronavirus detections, whether pools or individuals, on dates with at least one detection. In total, 2,529 fecal samples were tested across the five roosts sampled.

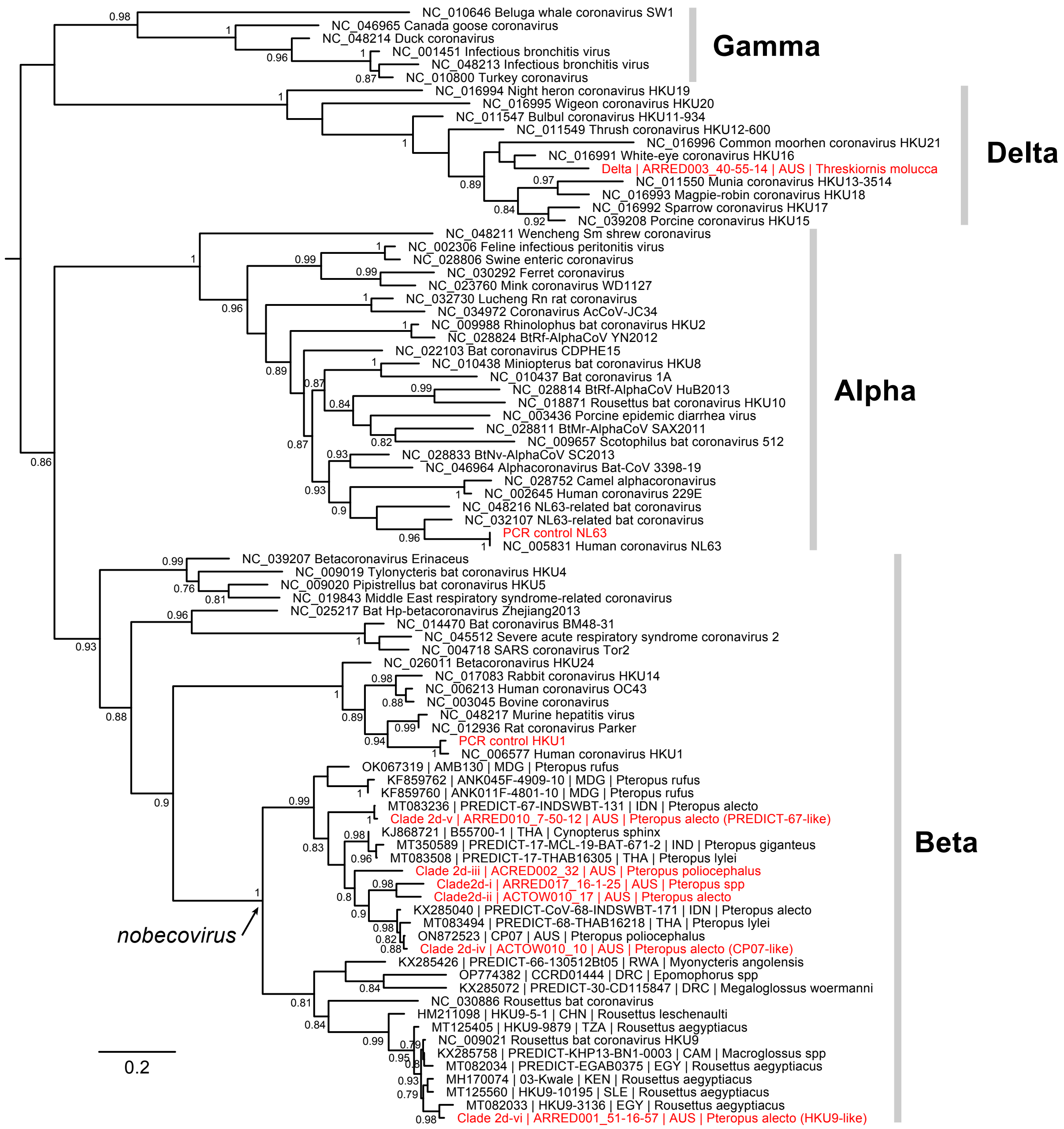

SI Figure 6. Phylogenetic analysis of all coronaviruses identified. Study sequences and select NCBI GenBank reference strains spanning the target RdRp region (390nt - ORF1b) were codon aligned and analyzed using a maximum likelihood approach. The coronaviruses identified in this study are highlighted with red colored text. Diversity of all four major coronavirus groups (alpha, beta, delta and gamma) are provided with the branch of the nobecovirus subgenus indicated with a pointed arrow. Branch support is indicated at node with SH-like values (>0.75) and all branches are scaled to the number of substitutions per site.

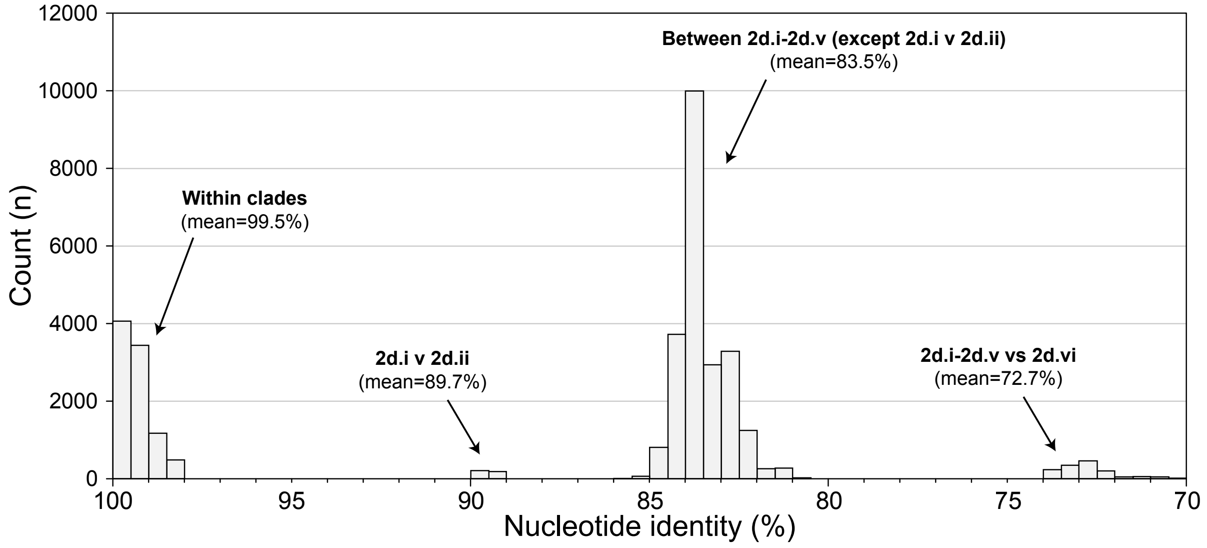

SI Figure 7. Pairwise-distances within and between coronavirus clades. All study sequences were codon aligned before the pairwise (nucleotide) distance was compared. The resultant matrix was examined by binning the distances into 0.5% increments and plotting distance (x-axis, nucleotide identity %) and counts (y-axis, n). The mean nucleotide identity for select clusters (within and between clades) has been provided.

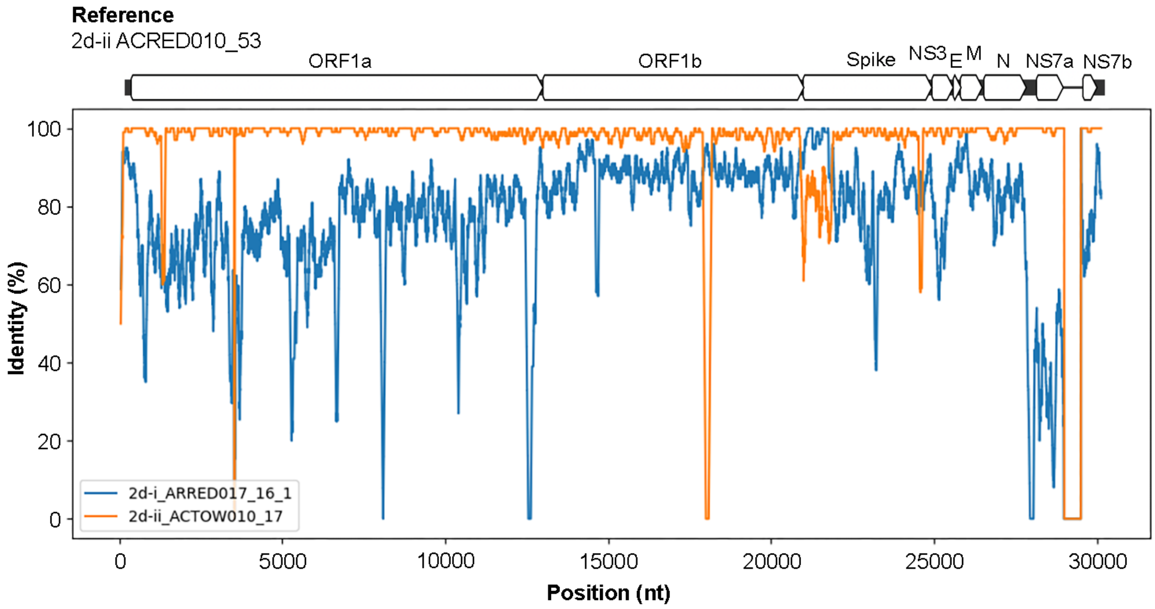

SI Figure 8. Similarity plot analysis of putative recombinant strain 2d-ii ACRED010_53. A mosaic insertion in the N-terminal region of the spike protein is evident when using the parental strains 2d-i ARRED017_16_1 (blue line) and 2d-ii ACTOW010_17 (orange line). The vertical axis represents the shared percent nucleotide identity between the putative recombinant and each parental strain The horizontal axis shows the nucleotide position along the full-length genome.

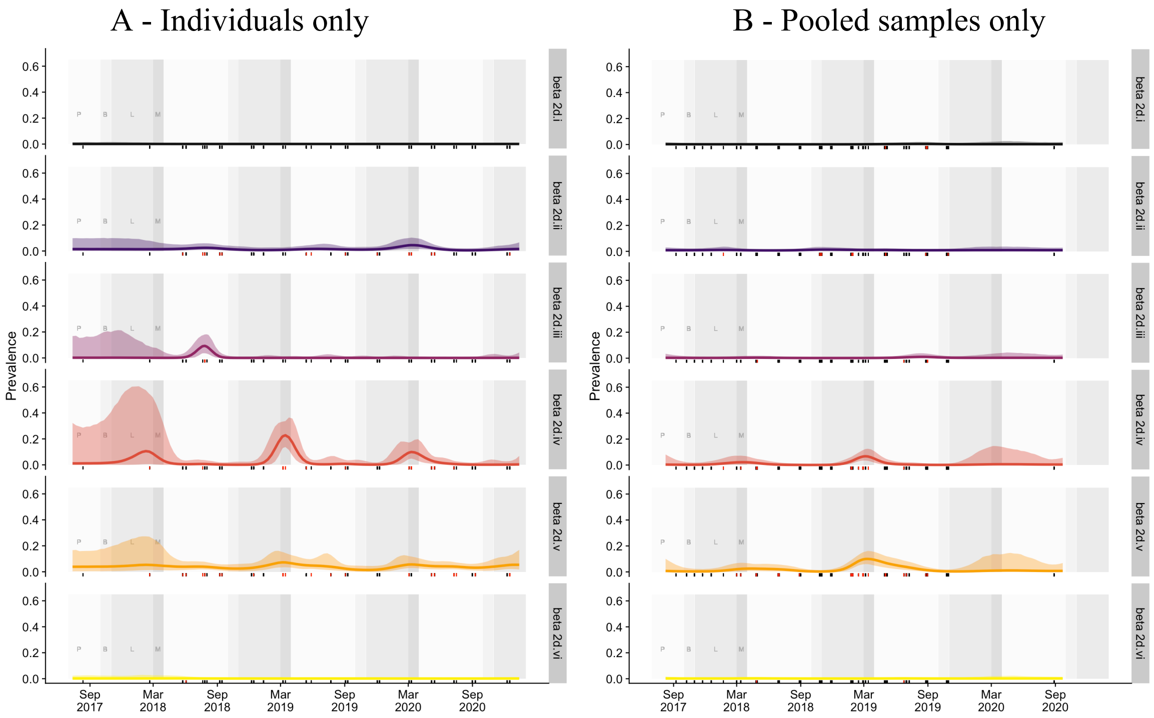

SI Figure 9. Time-dependent shedding patterns at the population level for the six coronavirus clades identified, showing results from individual samples alone (A) and pooled samples alone (B), compared to the model using integration of both individual and population level information (Main text Figure 2). The background shading corresponds to annual cycles associated with bat behavior where P is pregnancy, B is birth, L is lactation, and M is mating. The solid lines correspond to the posterior mean and the colored bands are the 95% credible intervals. The circles correspond to the unique sampling sessions (y axis = prevalence, size = number of samples (individuals) or pools (under-roost), open circles = samples from individual bats (Ind), closed circles = under-roost pooled samples (UR)).

Credible intervals are wider for individual samples (A) or pooled samples (B) alone, compared to the more precise credible intervals obtained with the integrated data (Figure 2), corresponding to time periods where fewer samples of that type were tested. In March 2019 the combined data from both individuals and pools (Figure 2), results in a higher prevalence estimate for clade 2d.iv than would be derived from the pools alone, while providing a more precise interval than either individuals (A) or pools (B) alone. While the true prevalence in this population remains unknown, previous simulation results[1] suggests that this combined prevalence estimate more closely represents true prevalence than either individual or pooled estimates alone.

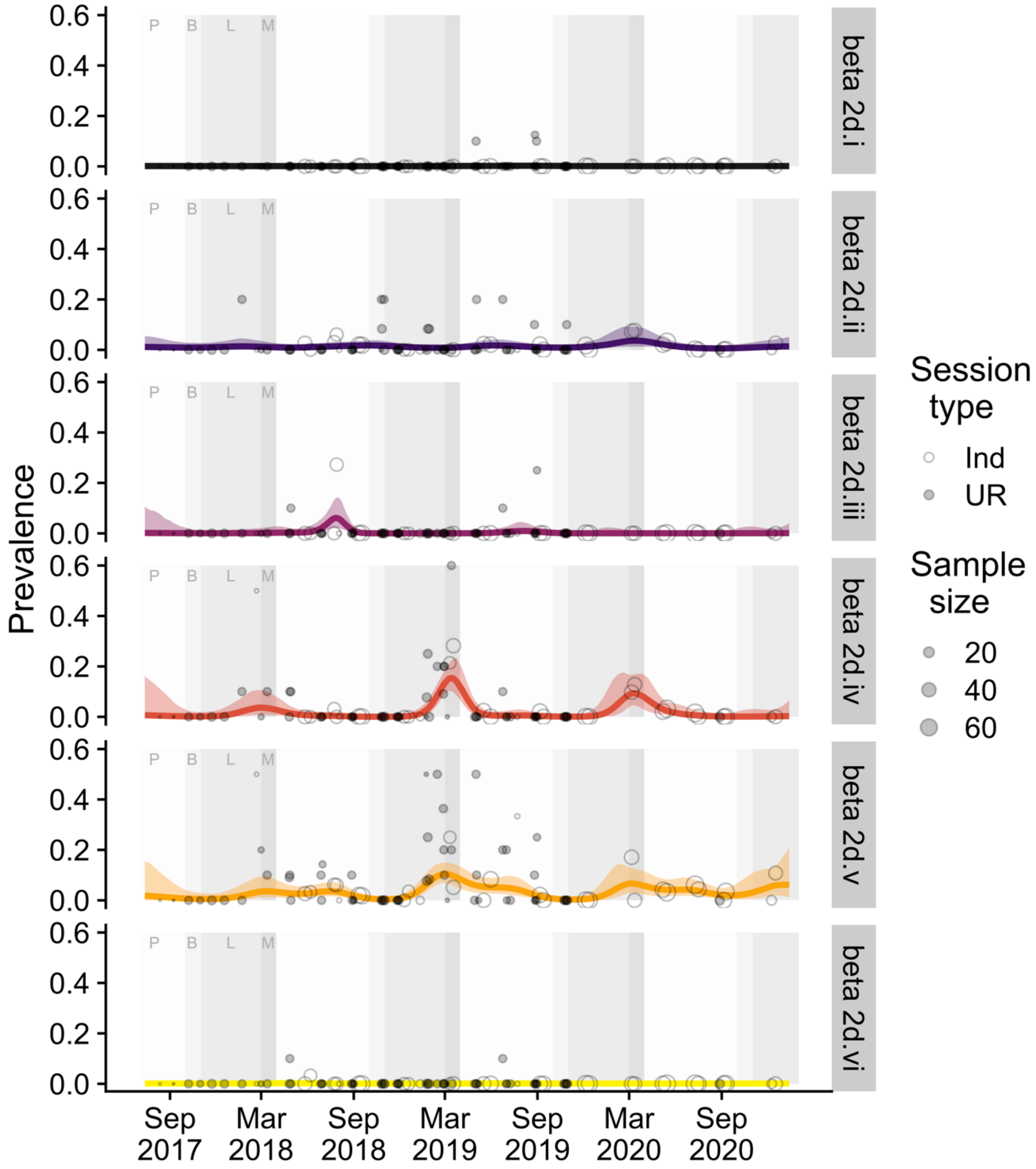

SI Figure 10. Time-dependent shedding patterns at the population level for the six coronavirus clades identified, based on the most supported model using integration of both individual and population level information. The background shading corresponds to annual cycles associated with bat behavior where P is pregnancy, B is birth, L is lactation, and M is mating. The solid lines correspond to the posterior mean and the colored bands are the 95% credible intervals. The circles correspond to the unique sampling sessions (y axis = prevalence, size = number of samples (individuals) or pools (under-roost), open circles = samples from individual bats (Ind), closed circles = under-roost pooled samples (UR)).

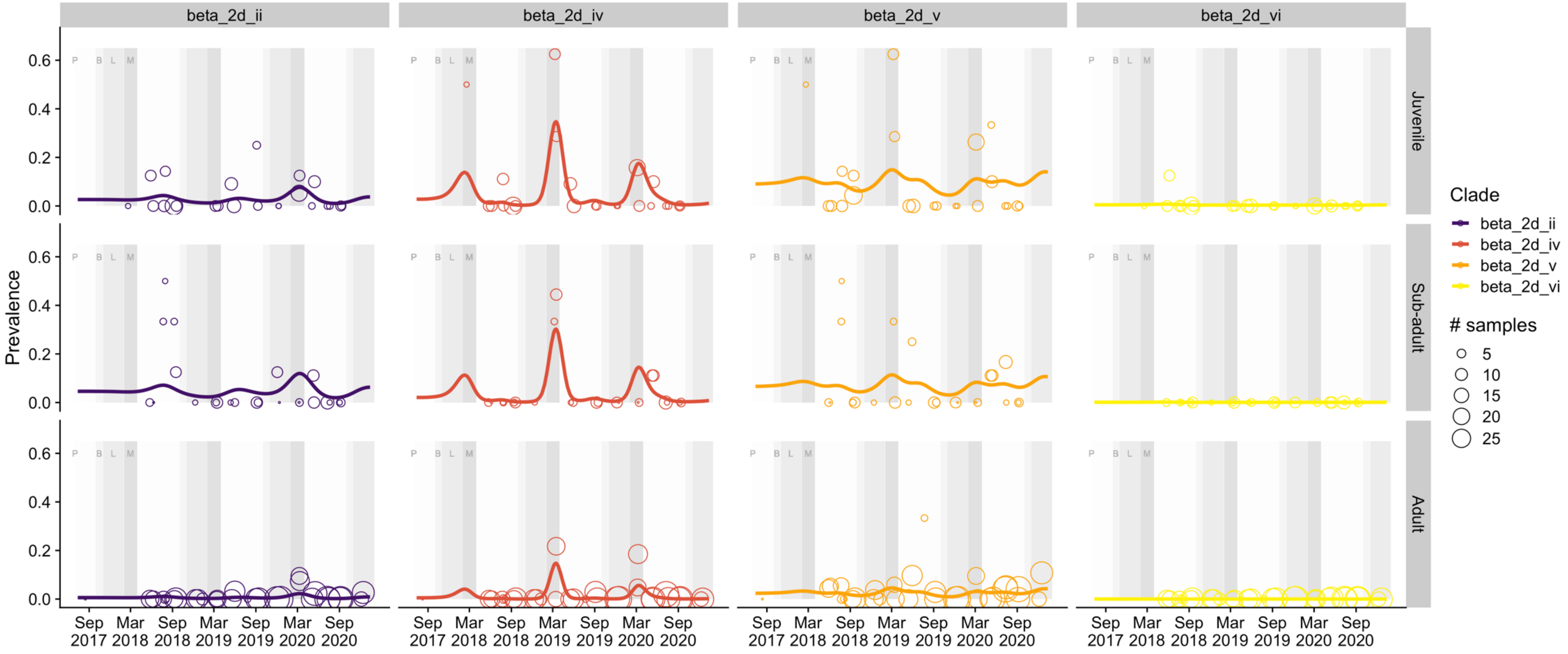

SI Figure 11. Individual-level coronavirus prevalence dynamics (using individual data only), as per Figure 4C, with background shading corresponding to annual reproductive cycles to facilitate assessment of seasonality (P is pregnancy, B is births, L is lactation, and M is mating). Juveniles were not typically sampled during the lactation period. Two individuals with missing age data are omitted.

SI Table 1. Catching sampling effort, indicating the total number of samples tested from each session, species and age class (Total individuals: 1137). Dates are in dd/mm/yyyy format. *Totals from sessions marked with an asterisk each include one additional individual for which an age class was not assigned.

Site	Start Date	End Date	Species	Adults	Juveniles	Subadults	Total
Clunes	12/8/2017	12/8/2017	*Pteropus alecto gouldi*	1	0	0	1
Clunes	19/2/2018	19/2/2018	*Pteropus alecto gouldi*	0	2	0	2
Clunes	2/8/2018	2/8/2018	*Pteropus alecto gouldi*	3	0	0	3
Redcliffe	25/5/2018	25/5/2018	*Pteropus alecto gouldi*	23	8	4	35
Redcliffe	25/5/2018	25/5/2018	*Pteropus poliocephalus*	0	3	0	3
Redcliffe	27/7/2018	27/7/2018	*Pteropus alecto gouldi*	5	7	2	14
Redcliffe	27/7/2018	27/7/2018	*Pteropus poliocephalus*	2	14	3	19
Redcliffe	14/9/2018	14/9/2018	*Pteropus alecto gouldi*	38	8	8	54
Redcliffe	14/12/2018	14/12/2018	*Pteropus alecto gouldi*	27	0	0	27
Redcliffe	14/12/2018	14/12/2018	*Pteropus poliocephalus*	1	0	0	1
Redcliffe	8/3/2019	8/3/2019	*Pteropus alecto gouldi*	17	8	3	28
Redcliffe	28/5/2019	28/5/2019	*Pteropus alecto gouldi*	31	13	4	48
Redcliffe	10/9/2019	10/9/2019	*Pteropus alecto gouldi*	38	5	4	47
Redcliffe	3/12/2019	6/12/2019	*Pteropus alecto gouldi*	48	2	8	58
Redcliffe	3/3/2020	3/3/2020	*Pteropus alecto gouldi*	21	19	1	41
Redcliffe	11/5/2020	15/5/2020	*Pteropus alecto gouldi*	44	10	9	63
Redcliffe	7/7/2020	10/7/2020	*Pteropus alecto gouldi*	50	3	12	65
Redcliffe	7/9/2020	9/9/2020	*Pteropus alecto gouldi*	49	4	5	58
Redcliffe	15/12/2020	17/12/2020	*Pteropus alecto gouldi*	37	0	0	37
Toowoomba	3/6/2018	3/6/2018	*Pteropus alecto gouldi*	19	8	1	29*
Toowoomba	3/6/2018	3/6/2018	*Pteropus poliocephalus*	0	1	0	1
Toowoomba	21/7/2018	21/7/2018	*Pteropus alecto gouldi*	18	9	3	30
Toowoomba	21/7/2018	21/7/2018	*Pteropus poliocephalus*	0	2	0	2
Toowoomba	8/9/2018	8/9/2018	*Pteropus alecto gouldi*	21	23	3	47
Toowoomba	8/9/2018	8/9/2018	*Pteropus poliocephalus*	0	2	0	2
Toowoomba	8/12/2018	8/12/2018	*Pteropus alecto gouldi*	27	0	2	29
Toowoomba	8/12/2018	8/12/2018	*Pteropus poliocephalus*	1	0	0	1
Toowoomba	11/1/2019	11/1/2019	*Pteropus alecto gouldi*	10	0	0	10
Toowoomba	15/3/2019	15/3/2019	*Pteropus alecto gouldi*	23	7	9	39
Toowoomba	14/5/2019	14/5/2019	*Pteropus alecto gouldi*	27	11	2	40
Toowoomba	23/7/2019	23/7/2019	*Pteropus alecto gouldi*	3	0	0	3
Toowoomba	3/9/2019	3/9/2019	*Pteropus alecto gouldi*	34	4	9	47
Toowoomba	10/12/2019	10/12/2019	*Pteropus alecto gouldi*	50	2	1	53
Toowoomba	10/3/2020	10/3/2020	*Pteropus alecto gouldi*	27	8	4	39
Toowoomba	4/5/2020	7/5/2020	*Pteropus alecto gouldi*	36	3	9	48
Toowoomba	14/7/2020	17/7/2020	*Pteropus alecto gouldi*	41	3	2	46
Toowoomba	1/9/2020	1/9/2020	*Pteropus alecto gouldi*	42	7	2	51
Toowoomba	8/12/2020	9/12/2020	*Pteropus alecto gouldi*	15	0	0	16*

SI Table 2. Underroost sampling effort, indicating the total number of samples from each session, their pooling rate for PCR screening (Total 1392 fecal samples across 510 pools). Dates are in dd/mm/yyyy format. Species recorded present in the roost at the time of sampling: BFF (*Pteropus alecto gouldi*), GHFF: *P. poliocephalus*; LRFF: *P. scapulatus*)

Site	Date	***Species present at roost***	Total number of pools	Total number of samples
Burleigh	1/7/2018	BFF	7	20
Burleigh	31/8/2018	BFF	4	12
Burleigh	25/10/2018	BFF, GHFF	2	5
Burleigh	23/1/2019	BFF	2	6
Burleigh	6/3/2019	BFF	2	3
Burleigh	28/8/2019	BFF, GHFF	4	11
Burleigh	30/10/2019	BFF, GHFF	12	30
Clunes	1/11/2018	BFF, GHFF	13	30
Clunes	29/11/2018	BFF, GHFF	8	23
Clunes	26/1/2019	BFF, GHFF	12	28
Clunes	27/2/2019	BFF, GHFF	10	28
Clunes	7/5/2019	BFF, GHFF	5	15
Clunes	2/7/2019	BFF, GHFF	7	12
Clunes	31/8/2019	BFF, GHFF	8	17
Clunes	24/10/2019	BFF, GHFF	11	30
Clunes	28/8/2020	BFF, GHFF	14	23
Redcliffe	27/4/2018	BFF, GHFF, LRFF	10	30
Redcliffe	29/6/2018	BFF, GHFF	10	30
Redcliffe	28/8/2018	BFF, GHFF	10	30
Redcliffe	31/10/2018	BFF	10	30
Redcliffe	30/11/2018	BFF, GHFF	10	30
Redcliffe	14/2/2019	BFF	10	30
Redcliffe	28/2/2019	BFF	10	30
Redcliffe	29/4/2019	BFF, LRFF	10	30
Redcliffe	9/7/2019	BFF	10	27
Redcliffe	30/8/2019	BFF, GHFF	10	30
Redcliffe	25/10/2019	BFF, GHFF	10	30
Sunnybank	8/9/2017	BFF, GHFF	1	1
Sunnybank	8/10/2017	BFF, GHFF	10	30
Sunnybank	31/10/2017	BFF, GHFF	7	20
Sunnybank	23/11/2017	BFF, GHFF	12	30
Sunnybank	18/12/2017	BFF, GHFF	11	30
Sunnybank	22/1/2018	BFF, GHFF	10	30
Sunnybank	1/3/2018	BFF, GHFF	5	14
Sunnybank	13/3/2018	BFF, GHFF	10	30
Sunnybank	26/4/2018	BFF	11	30
Sunnybank	1/9/2018	BFF	7	21
Sunnybank	27/10/2018	BFF, GHFF	12	30
Sunnybank	26/11/2018	BFF, GHFF	9	26
Sunnybank	24/1/2019	BFF, GHFF	13	30
Sunnybank	26/2/2019	BFF, GHFF, LRFF	11	30
Sunnybank	3/5/2019	BFF, GHFF	10	20
Sunnybank	1/7/2019	BFF, GHFF	10	22
Sunnybank	27/8/2019	BFF, GHFF	8	22
Sunnybank	28/10/2019	BFF, GHFF	10	30
Toowoomba	29/4/2018	BFF, GHFF	10	30
Toowoomba	28/6/2018	BFF, GHFF	10	28
Toowoomba	29/8/2018	BFF, GHFF	10	30
Toowoomba	26/10/2018	BFF, GHFF	10	30
Toowoomba	27/11/2018	BFF	10	30
Toowoomba	29/1/2019	BFF, GHFF	12	30
Toowoomba	14/3/2019	BFF, GHFF, LRFF	10	30
Toowoomba	2/5/2019	BFF, GHFF	10	30
Toowoomba	24/6/2019	BFF, GHFF	10	30
Toowoomba	26/8/2019	BFF, GHFF	10	30
Toowoomba	29/10/2019	BFF, GHFF, LRFF	10	30

SI Table 3. Full details of the genome sequences obtained.

Strain	RdRp-clade	Sampling site	Country	Location coordinates	Collection date	Sampling type	Pooled source	Host observed	Host COXI*	GenBank	SRA
ARRED017_16_1	2d-i	QLD: Redcliffe	Australia	27.23°S, 153.10°E	2019-08-30	Under roost	No	Pteropus alecto	Pteropus alecto (71.9%); Pteropus conspicillatus (23.3%); Pteropus spp (4.8%)	PV683367	SRR33676035
ACTOW010_17	2d-ii	QLD: Toowoomba	Australia	27.60°S, 151.94°E	2019-09-04	Individual	No	Pteropus alecto	Pteropus alecto (95.9%); Pteropus scapulatus (4.1%)	PV683362	SRR33676034
ACRED010_53	2d-ii	QLD: Redcliffe	Australia	27.23°S, 153.10°E	2020-03-06	Individual	No	Pteropus alecto	Pteropus alecto (93.8%); Pteropus scapulatus (4.0%); Pteropus spp (1.5%); Geotrygon spp (0.7%)	PV683361	SRR33676033
ACRED002_71	2d-iii	QLD: Redcliffe	Australia	27.23°S, 153.10°E	2018-07-29	Individual	No	Pteropus poliocephalus	Pteropus poliocephalus (66.5%); Pteropus alecto (14.0%); Pteropus scapulatus (8.6%); Pteropus spp (10.5%); Thrips spp (0.4%)	PV683360	SRR33676032
ACRED002_7	2d-iii	QLD: Redcliffe	Australia	27.23°S, 153.10°E	2018-07-27	Individual	No	Pteropus poliocephalus	Thrips spp (82.4%); Pteropus poliocephalus (12.6%); Pteropus alecto (2.8%); Pteropus spp (2.2%)	PV683359	SRR33676031
ARCLU022	2d-iv	NSW: Clunes	Australia	28.73°S, 153.42°E	2019-10-24	Under roost	Yes	Pteropus spp		PV683363	SRR33676030
CP07	2d-iv	NSW: Centennial Park	Australia	33. 90°S, 151.24°E	2019-02-26	Under roost	Yes	Pteropus poliocephalus	N/A	ON872523	SRR19790900
ARRED010	2d-v	QLD: Redcliffe	Australia	27.23°S, 153.10°E	2019-02-14	Under roost	Yes	Pteropus spp		PV683365	SRR33676029
ARRED010_7-50-12	2d-v	QLD: Redcliffe	Australia	27.23°S, 153.10°E	2019-02-14	Under roost	Yes	Pteropus alecto	Pteropus alecto (79.7%); Pteropus conspicillatus (13.0%); Pteropus tonganus (4.2%); Pteropus scapulatus (1.3%); Pteropus spp (1.9%)	PV683366	SRR33676028

AMB130	2d	Ambanja: Ambakoana	Madagascar	21.00°S, 47.97°E	2018-02-26	Individual	No	Pteropus rufus	N/A	OK067319	N/A
ARRED001_51-16-57	2d-vi	QLD: Redcliffe	Australia	27.23°S, 153.10°E	2018-04-21	Under roost	Yes	Pteropus alecto	Pteropus alecto (74.5%); Pteropus conspicillatus (20.6%); Pteropus spp (2.5%); Pteropus tonganus (2.0%); Pteropus scapulatus (0.4%)	PV683364	SRR33676027

*Introgression and hybridisaiton is well recognised within *Pteropus* spp[2], including P. alecto, P. poliocephalus and P conspicillatus (species present within Australia) and also *P. griseus* (Indonesia)[3].

SI Table 4. Recombination analysis using genome sequences.

Putative recombinant	Parent 1	Parent 2	Genome position (nt)		Recombination Detection Program 4 method (p-value)						
			Start	End	RDP	GENECONV	Bootscan	Maxchi	Chimaera	SiSscan	3Seq
Strain 2d-ii ACRED010_53	Clade 2d-ii	Clade 2d-i	20396	21269	5.44E-163	7.22E-141	3.93E-136	2.61E-25	2.15E-25	5.66E-33	6.06E-13
Clade 2d-iii	Clade 2d-iv	Clade 2d-v	20505	25907	6.98E-26	4.00E-06	2.35E-30	9.47E-09	3.74E-05	1.49E-43	2.31E-14
Clade 2d-ii	Clade 2d-v	Clade 2d-i	13768	27350	9.96E-87	Not significant	1.64E-116	3.06E-61	1.33E-313	3.63E-69	5.96E-14

SI Table 5. Prevalence of coronavirus clades in individuals by species and age class. Two individual black flying foxes did not have an age assigned at during sampling, and were both negative across all clades.

Clade	Species	Age	Number positive	Total	Prevalence (%)
2d.i	*Pteropus alecto gouldi*	Juvenile	0	174	0
2d.i	*Pteropus alecto gouldi*	Subadult	0	107	0
2d.i	*Pteropus alecto gouldi*	Adult	0	825	0
2d.ii	*Pteropus alecto gouldi*	Juvenile	7	174	4
2d.ii	*Pteropus alecto gouldi*	Subadult	6	107	5.6
2d.ii	*Pteropus alecto gouldi*	Adult	7	825	0.8
2d.iii	*Pteropus alecto gouldi*	Juvenile	0	174	0
2d.iii	*Pteropus alecto gouldi*	Subadult	0	107	0
2d.iii	*Pteropus alecto gouldi*	Adult	0	825	0
2d.iv	*Pteropus alecto gouldi*	Juvenile	14	174	8
2d.iv	*Pteropus alecto gouldi*	Subadult	7	107	6.5
2d.iv	*Pteropus alecto gouldi*	Adult	13	825	1.6
2d.v	*Pteropus alecto gouldi*	Juvenile	17	174	9.8
2d.v	*Pteropus alecto gouldi*	Subadult	8	107	7.5
2d.v	*Pteropus alecto gouldi*	Adult	22	825	2.7
2d.vi	*Pteropus alecto gouldi*	Juvenile	1	174	0.6
2d.vi	*Pteropus alecto gouldi*	Subadult	0	107	0
2d.vi	*Pteropus alecto gouldi*	Adult	0	825	0
2d.i	*Pteropus poliocephalus*	Juvenile	0	22	0
2d.i	*Pteropus poliocephalus*	Subadult	0	3	0
2d.i	*Pteropus poliocephalus*	Adult	0	4	0
2d.ii	*Pteropus poliocephalus*	Juvenile	0	22	0
2d.ii	*Pteropus poliocephalus*	Subadult	0	3	0
2d.ii	*Pteropus poliocephalus*	Adult	0	4	0
2d.iii	*Pteropus poliocephalus*	Juvenile	5	22	22.7
2d.iii	*Pteropus poliocephalus*	Subadult	3	3	100
2d.iii	*Pteropus poliocephalus*	Adult	1	4	25
2d.iv	*Pteropus poliocephalus*	Juvenile	0	22	0
2d.iv	*Pteropus poliocephalus*	Subadult	0	3	0
2d.iv	*Pteropus poliocephalus*	Adult	0	4	0
2d.v	*Pteropus poliocephalus*	Juvenile	0	22	0
2d.v	*Pteropus poliocephalus*	Subadult	0	3	0
2d.v	*Pteropus poliocephalus*	Adult	0	4	0
2d.vi	*Pteropus poliocephalus*	Juvenile	0	22	0
2d.vi	*Pteropus poliocephalus*	Subadult	0	3	0
2d.vi	*Pteropus poliocephalus*	Adult	0	4	0

SI Table 6. Details of recaptured individuals where fecal samples were tested across multiple capture events.

Site	Capture date	Recapture date	Species	Sex	Age	Result
Redcliffe	10/9/2019	3/12/2019	*Pteropus alecto gouldi*	Female	Adult	Negative
Redcliffe	10/9/2019	15/5/2020	*Pteropus alecto gouldi*	Female	Adult	Negative
Redcliffe	10/9/2019	15/5/2020	*Pteropus alecto gouldi*	Male	Adult	Negative
Redcliffe	10/9/2019	15/5/2020	*Pteropus alecto gouldi*	Male	Adult	Negative
Redcliffe	10/9/2019	17/12/2020	*Pteropus alecto gouldi*	Female	Adult	Negative
Redcliffe	3/12/2019	15/5/2020	*Pteropus alecto gouldi*	Male	Adult	Negative then 2d.ii pos
Redcliffe	3/12/2019	15/5/2020	*Pteropus alecto gouldi*	Male	Adult	Negative
Redcliffe	3/12/2019	17/12/2020	*Pteropus alecto gouldi*	Female	Adult	Negative
Toowoomba	10/12/2019	7/5/2020	*Pteropus alecto gouldi*	Male	Adult	Negative
Toowoomba	10/12/2019	17/7/2020	*Pteropus alecto gouldi*	Female	Adult	Negative
Redcliffe	3/3/2020	10/7/2020	*Pteropus alecto gouldi*	Female	Subadult	2d.v pos then Negative

Supplementary references

[1]Hoegh, A., Peel, A.J., Madden, W., Aravena, M.R., Morris, A., Washburne, A. & Plowright, R.K. (2021). Estimating viral prevalence with data fusion for adaptive two-phase pooled sampling. *Ecol Evol.*

[2]Neaves, L.E., Danks, M., Lott, M.J., Dennison, S., Frankham, G.J., King, A., Eldridge, M.D.B., Johnson, R.N. & Divljan, A. (2018). Unmasking the complexity of species identification in Australasian flying-foxes. *Plos One* **13**, e0194908.

[3]Peel, A.J., Yinda, C.K., Annand, E.J., Dale, A.S., Eby, P., Eden, J.-S., Jones, D.N., Kessler, M.K., Lunn, T.J., Pearson, T., Schulz, J.E., Smith, I.L., Munster, V.J., Plowright, R.K. & Group, B.O.H. (2022). Novel Hendra Virus Variant Circulating in Black Flying Foxes and Grey-Headed Flying Foxes, Australia. *Emerg. Infect. Dis.* **28**, 1043–1047.
