# Supplementary material for: Synchronized seasonal excretion of multiple coronaviruses coincides with high rates of coinfection in immature bats
Source: Nat Commun. 2025 Jul 17;16:6579. doi: 10.1038/s41467-025-61521-7 (PMC12271399; doi:10.1038/s41467-025-61521-7)
Supplement: Supplementary file 2 — Reporting Summary [file 41467_2025_61521_MOESM2_ESM.pdf]

nature portfolio

Corresponding author(s): Andrew Hoegh, John-Sebastian Eden, Raina K. Plowright: rkp57@cornell.edu; js.eden@sydney.edu.au; andrew.hoegh@montana.edu

Last updated by author(s): Jun 23, 2025

Reporting Summary

Nature Portfolio wishes to improve the reproducibility of the work that we publish. This form provides structure for consistency and transparency in reporting. For further information on Nature Portfolio policies, see our Editorial Policies and the Editorial Policy Checklist.

Statistics

For all statistical analyses, confirm that the following items are present in the figure legend, table legend, main text, or Methods section.

n/a	Confirmed	
☐	☒	The exact sample size (*n*) for each experimental group/condition, given as a discrete number and unit of measurement
☐	☒	A statement on whether measurements were taken from distinct samples or whether the same sample was measured repeatedly
☒	☐	The statistical test(s) used AND whether they are one- or two-sided<br>*Only common tests should be described solely by name; describe more complex techniques in the Methods section.*
☐	☒	A description of all covariates tested
☐	☒	A description of any assumptions or corrections, such as tests of normality and adjustment for multiple comparisons
☐	☒	A full description of the statistical parameters including central tendency (e.g. means) or other basic estimates (e.g. regression coefficient) AND variation (e.g. standard deviation) or associated estimates of uncertainty (e.g. confidence intervals)
☐	☒	For null hypothesis testing, the test statistic (e.g. *F*, *t*, *r*) with confidence intervals, effect sizes, degrees of freedom and *P* value noted<br>*Give P values as exact values whenever suitable.*
☐	☒	For Bayesian analysis, information on the choice of priors and Markov chain Monte Carlo settings
☒	☐	For hierarchical and complex designs, identification of the appropriate level for tests and full reporting of outcomes
☐	☒	Estimates of effect sizes (e.g. Cohen's *d*, Pearson's *r*), indicating how they were calculated

Our web collection on statistics for biologists contains articles on many of the points above.

Software and code

Policy information about availability of computer code

Data collection	N/A
Data analysis	Code generated during the current study, and the input data for the models and figures, are available at: https://zenodo.org/records/15626080. This analysis used version 4.4.1 of R and version 2.32.2 of stan.

For manuscripts utilizing custom algorithms or software that are central to the research but not yet described in published literature, software must be made available to editors and reviewers. We strongly encourage code deposition in a community repository (e.g. GitHub). See the Nature Portfolio guidelines for submitting code & software for further information.

Data

Policy information about availability of data

All manuscripts must include a data availability statement. This statement should provide the following information, where applicable:

- Accession codes, unique identifiers, or web links for publicly available datasets
- A description of any restrictions on data availability
- For clinical datasets or third party data, please ensure that the statement adheres to our policy

The field data and CoV clade detection data generated and analysed in this study have been deposited in the Cornell University eCommons Digital Repository, with the DOI 10.7298/w7sw-6161, available at: https://doi.org/10.7298/w7sw-6161. The combined processed data used as input for models and figures are available at https://zenodo.org/records/15626080, with the relevant code. Sequences used and generated in this study are available in online repositories as per links below.

Additional details are provided in SI Table 3.
Existing GenBank sequences: ON872523 [https://www.ncbi.nlm.nih.gov/nuccore/ON872523], OK067319 [https://www.ncbi.nlm.nih.gov/nuccore/OK067319].
New GenBank sequences: PV683367 [https://www.ncbi.nlm.nih.gov/nuccore/PV683367], PV683362 [https://www.ncbi.nlm.nih.gov/nuccore/PV683362], PV683361 [https://www.ncbi.nlm.nih.gov/nuccore/PV683361], PV683360 [https://www.ncbi.nlm.nih.gov/nuccore/PV683360], PV683359 [https://www.ncbi.nlm.nih.gov/nuccore/PV683359], PV683363 [https://www.ncbi.nlm.nih.gov/nuccore/PV683363], PV683365 [https://www.ncbi.nlm.nih.gov/nuccore/PV683365], PV683366 [https://www.ncbi.nlm.nih.gov/nuccore/PV683366], PV683364 [https://www.ncbi.nlm.nih.gov/nuccore/PV683364].
New SRA sequences: SRR33676035 [https://www.ncbi.nlm.nih.gov/nuccore/SRR33676035], SRR33676034 [https://www.ncbi.nlm.nih.gov/nuccore/SRR33676034], SRR33676033 [https://www.ncbi.nlm.nih.gov/nuccore/SRR33676033], SRR33676032 [https://www.ncbi.nlm.nih.gov/nuccore/SRR33676032], SRR33676031 [https://www.ncbi.nlm.nih.gov/nuccore/SRR33676031], SRR33676030 [https://www.ncbi.nlm.nih.gov/nuccore/SRR33676030], SRR19790900 [https://www.ncbi.nlm.nih.gov/nuccore/SRR19790900], SRR33676029 [https://www.ncbi.nlm.nih.gov/nuccore/SRR33676029], SRR33676028 [https://www.ncbi.nlm.nih.gov/nuccore/SRR33676028], SRR33676027 [https://www.ncbi.nlm.nih.gov/nuccore/SRR33676027],

Research involving human participants, their data, or biological material

Policy information about studies with human participants or human data. See also policy information about sex, gender (identity/presentation), and sexual orientation and race, ethnicity and racism.

Reporting on sex and gender	N/A
Reporting on race, ethnicity, or other socially relevant groupings	N/A
Population characteristics	N/A
Recruitment	N/A
Ethics oversight	N/A

Note that full information on the approval of the study protocol must also be provided in the manuscript.

Field-specific reporting

Please select the one below that is the best fit for your research. If you are not sure, read the appropriate sections before making your selection.

☐ Life sciences ☐ Behavioural & social sciences ☒ Ecological, evolutionary & environmental sciences

For a reference copy of the document with all sections, see nature.com/documents/nr-reporting-summary-flat.pdf

Ecological, evolutionary & environmental sciences study design

All studies must disclose on these points even when the disclosure is negative.

Study description	The study investigates ecological interactions among bats, focusing on longitudinal sampling of wild bat populations, estimation of prevalence, and investigation of viral co-infections. It uses quantitative data, with units being faecal samples from individual bats and pooled samples collected from underneath bat roosts.
Research sample	The sampling targeted Australian flying fox populations (specifically, black flying foxes Pteropus alecto and grey-headed flying fox Pteropus poliocephalus) in southeast Queensland and Northeast New South Wales. Sampling site selection was based on roost conditions known to be important for viral spillover risk (continuous occupancy during winter, recently formed, occupied by black flying foxes). Within each site, sample selection is random, following established protocols.
Sampling strategy	Sample sizes were predetermined based on expected viral prevalence and statistical power calculations.
Data collection	Data were collected via field sampling of bat populations, molecular viral screening assays and sequencing. Field samples were handled by trained field teams, with data collection occurring either on paper data sheets, transferred to digital formats, or directly onto a tablet data collection form.
Timing and spatial scale	Field data were collected monthly over three consecutive years at five roosting sites across southeastern Australia. Laboratory data were gathered continuously over the course of the study.
Data exclusions	Two individuals which did not have an age class recorded were excluded from age-based individual analyses
Reproducibility	Because this is a field study in wild populations, results cannot be directly replicated. However, findings were replicated across sites and years.
Randomization	Individuals were captured in nets at the roost site. All captured individuals that produced a faecal sample during holding or processing were included in screening and analyses. Multiple faecal samples were collected from each under-roost sheet, randomly selected from available samples. A subset of under-roost faecal samples were randomly selected for pooling and viral screening. collected from.

Blinding	The laboratory team was blind to the metadata associated with each sample.

Did the study involve field work? ☒ Yes ☐ No

Field work, collection and transport

Field conditions	Fieldwork was conducted across all seasons and under varying climatic conditions, however rain-affected sampling sessions were abandoned and rescheduled.
Location	Sampling occurred in southeastern Australia, in roost sites located between 27.6°S and 28.7°S latitude (Toowoomba (-27.60 S, 151.94 E), Redcliffe (-27.23 S, 153.10 E), Sunnybank (-27.58 S, 153.05 E) and Burleigh Knoll (-28.08 S, 153.44 E), in Queensland, Australia and Clunes (-28.73 S, 153.42 E), New South Wales (NSW))
Access & import/export	All necessary permits for sample collection were obtained from relevant wildlife authorities. Samples were not exported.
Disturbance	Disturbance to bat populations was minimized by conducting individual sampling outside of the birthing and early lactation season and limiting the time of interaction with captured animals.

Reporting for specific materials, systems and methods

We require information from authors about some types of materials, experimental systems and methods used in many studies. Here, indicate whether each material, system or method listed is relevant to your study. If you are not sure if a list item applies to your research, read the appropriate section before selecting a response.

Materials & experimental systems

n/a	Involved in the study	
☒	☐	Antibodies
☒	☐	Eukaryotic cell lines
☒	☐	Palaeontology and archaeology
☐	☒	Animals and other organisms
☒	☐	Clinical data
☒	☐	Dual use research of concern
☒	☐	Plants

Methods

n/a	Involved in the study	
☒	☐	ChIP-seq
☒	☐	Flow cytometry
☒	☐	MRI-based neuroimaging

Animals and other research organisms

Policy information about studies involving animals; ARRIVE guidelines recommended for reporting animal research, and Sex and Gender in Research

Laboratory animals	N/A
Wild animals	The study involved wild black flying foxes (Pteropus alecto; n = 1108) and grey-headed flying foxes (Pteropus poliocephalus; n= 29) and samples collected indirectly from underneath flying fox roosts (n = 1392). Bats were caught using mist nets and temporarily held before being sampled and released back into their roosts after sampling. No animals were harmed during the study. Animals were aged as juveniles (<12 months), subadults (12-24 months) or adults (>24 months), based on morphometrics and development of secondary sexual characteristics,
Reporting on sex	Sex was considered in the study design. Data were disaggregated by sex, and male and female bats were sampled and analyzed separately for reproductive status and age.
Field-collected samples	All samples were field-collected. Bat fecal samples were collected and stored at -20˚C or at -80˚C until laboratory analysis.
Ethics oversight	All animal handling was conducted under approval from the Griffith University Animal Ethics Committee (Certificate: ENV/10/16/AEC and ENV/07/20/AEC). Personal Protective Equipment and disinfection protocols followed best practice guidelines (e.g. IUCN Bat Specialist Group, 2021; Wildlife Health Australia, 2020).

Note that full information on the approval of the study protocol must also be provided in the manuscript.

Plants

Seed stocks	N/A
Novel plant genotypes	N/A
Authentication	N/A
